# Assessing the Severity of Traumatic Brain Injury—Time for a Change?

**DOI:** 10.3390/jcm10010148

**Published:** 2021-01-04

**Authors:** Olli Tenovuo, Ramon Diaz-Arrastia, Lee E. Goldstein, David J. Sharp, Joukje van der Naalt, Nathan D. Zasler

**Affiliations:** 1 Division of Clinical Neurosciences, Turku Brain Injury Centre, Turku University Hospital, 20521 Turku, Finland; 2 Department of Neurology, Institute of Clinical Medicine, University of Turku, 20500 Turku, Finland; 3 3 Perelman School of Medicine, University of Pennsylvania, Philadelphia, PA 19104, USA； ramon.diaz-arrastia@usuhs.edu; 4 Alzheimer's Disease Research Center, College of Engineering, Boston University School of Medicine, Boston, MA 02118, USA; lgold@bu.edu; 5 Clinical, cognitive and computational neuroimaging laboratory (C3NL), Department of Brain Sciences, Faculty of Medicine, Imperial College London, London, W12 0NN, UK; david.sharp@imperial.ac.uk; 6 UK Dementia Research Institute Care Research and Technology Centre, Imperial College London and the University of Surrey, London, W12 0NN UK; 7 Department of Physical Medicine and Rehabilitation, Virginia Commonwealth University, Richmond, VA 23284, USA; 8 Concussion Care Centre of Virginia and Tree of Life, Richmond, VA 23233, USA; nzasler@cccv-ltd.com; 9 Department of Physical Medicine and Rehabilitation, Virginia Commonwealth University, Richmond, VA 23284, USA

**Keywords:** traumatic brain injury, severity, assessment, outcome

## Abstract

Traumatic brain injury (TBI) has been described to be man’s most complex disease, in man’s most complex organ. Despite this vast complexity, variability, and individuality, we still classify the severity of TBI based on non-specific, often unreliable, and pathophysiologically poorly understood measures. Current classifications are primarily based on clinical evaluations, which are non-specific and poorly predictive of long-term disability. Brain imaging results have also been used, yet there are multiple ways of doing brain imaging, at different timepoints in this very dynamic injury. Severity itself is a vague concept. All prediction models based on combining variables that can be assessed during the acute phase have reached only modest predictive values for later outcome. Yet, these early labels of severity often determine how the patient is treated by the healthcare system at large. This opinion paper examines the problems and provides caveats regarding the use of current severity labels and the many practical and scientific issues that arise from doing so. The objective of this paper is to show the causes and consequences of current practice and propose a new approach based on risk classification. A new approach based on multimodal quantifiable data (including imaging and biomarkers) and risk-labels would be of benefit both for the patients and for TBI clinical research and should be a priority for international efforts in the field.

## 1. Introduction

Traumatic brain injuries (TBIs) are among the most common maladies affecting humanity. Grading injury severity is an early step in management and is a first step towards prognosis and targeted therapies. Fields of medicine as diverse as cardiology and oncology use grading schema, such as NYHA-congestive heart failure grading scale in cardiology and staging scales in many cancers. Grading based exclusively on clinical features, in the absence of imaging or molecular biomarkers, provides suboptimal prognostic information and is inadequate for identifying patients likely to benefit from specific therapies [1]. Grading scales that focus exclusively on clinical features may not capture disease endophenotypes or differentiate underlying disease mechanisms. Progress in clinical management has often been made when grading scales have incorporated measurable biomarkers directly related to the underlying pathophysiology, such as for choosing treatment options for gliomas or breast cancer based on biomarkers.

TBI has been historically classified as mild, moderate, and severe, based exclusively on clinical features such as the level of consciousness or duration of post-traumatic amnesia (PTA) [2]. The introduction of computerized tomography (CT) almost 40 years ago revolutionized the management of TBI, but primarily for the small subset (< 5%) of patients with TBI who require neurosurgical interventions. Over 90% of patients who sustain TBIs are classified in the mild end of the spectrum, including those injuries labeled as concussions. While most patients with a single mild TBI (mTBI) fully recover, some do not [3], leading to potentially prolonged suffering, impaired quality of life, and increased risk of post-traumatic sequelae. Because mTBI is so common, the cumulative social burden from mTBI approaches that arising from moderate to severe TBI [4], with treatment costs higher than those of more severe cases [5].

For decades, the cause of prolonged symptoms after mTBIs/concussions have been a subject of debate. This scientific confusion is reflected by the fact that mTBI has been defined more than 40 different ways in the scientific literature [6]. The situation is even more confusing for concussion, where there are no official or internationally agreed definitions [7]. The concept of concussion as a benign transient incident has prevailed until our times, and still strongly influences public opinion. However, recent work shows that even concussions can have long-lasting effects [8]. The inability to define concussion makes its use in clinical decision-making and research very problematic. There have been arguments made for classifying concussion as a type of mTBI [9]; yet, this position is not true in all guideline documents and, in practice, clinicians tend to use the terms mTBI and concussion synonymously. Studies in patients with “concussion” greatly overlap with studies in patients with mTBI, further confusing the field. In clinical reality, the ICD-10 diagnosis S06.0 (Commotio cerebri) is frequently used for patients with CT-negative TBI irrespective of the other clinical severity indices. These issues are of broad relevance as concerns about chronic traumatic encephalopathy (CTE) and other long-term sequelae of repetitive neurotrauma have spurred high-profile awareness campaigns, media discussions, and public health policies. In this discussion it often forgotten, that CTE seems to be more related to a large number of subconcussive impacts, than to the presence of a clearly demarcated TBI history.

In this paper, we will point out how the current tools for assessing the severity of TBI are far from ideal, and current methods of classifying severity include numerous sources of error. We will discuss how assigning seemingly definitive severity labels to patients following TBI often leads to confusion and problems for patients, families, caretakers, and health care systems. For neurotrauma research, these problems have the potential to lead to study variability and inconsistency of results. Finally, we will propose a new approach for classifying TBIs and what will be needed to build a new and better classification.

## 2. TBI Severity: What Does It Really Mean?

TBI severity can be assessed from several viewpoints (Table 1) and varies depending on who is making the evaluation. Available measures of biological injury are not currently used when assessing severity. Judgments about severity are context dependent, often subjective, and are influenced by the reference point and experience of the assessor. For example, a patient with life-threatening TBI surviving the critical early phase may recover to independent daily life, showing only mild deficits in the eyes of the neurosurgeon, but severe cognitive deficits assessed by the neuropsychologist, and profound emotional/personality changes by the family. This creates challenges, as outcomes after TBI are so variable. Injuries judged as severe in the acute setting may show full recovery, whereas those thought to be mild can lead to disabling consequences. We fully acknowledge that subjective views on outcome are not in any way specific to TBI, but simultaneously point out that few medical conditions are classified using such vaguely defined severity labels as in TBI. More importantly, because the whole concept of ’severity´ is so ill-defined, multifaceted and subjective, it should be abandoned and replaced with more appropriate descriptors, as discussed below.

## 3. Tools to Assess TBI Severity in the Acute Setting

Our current tools to assess the severity of TBI include level of consciousness, usually assessed with the Glasgow Coma Scale (GCS), and duration of PTA, assessed using tools such as the Galveston Orientation and Amnesia Test (GOAT) or Westmead PTA scale [10,11]. While the use of the GCS score has entered routine clinical practice, PTA is still rarely prospectively assessed in clinical settings. Regrettably, both the GCS score and the duration of PTA have been shown to be poorly aligned with pathophysiological substrates [12,13]. There are several regions of the brain that regulate consciousness and memory in a complex manner [14], and their neural basis is far from well understood.

### 3.1. Loss and Level of Consciousness

GCS was originally developed as a tool to assess level of consciousness, and not to predict the outcome of TBI or coma in general. It correlates poorly with clinical outcome following TBI for many reasons [15]. In most clinical settings, estimating the level of consciousness is routinely done using either the GCS or some other tool, but the context is very variable (e.g., time from the incident, presence of tracheostomy, training of the evaluator, state of other vital functions, and other potential confounders) making the use and interpretation complex. A GCS score of 5 some minutes after the injury has different significance than one recorded hours or days after the injury. GCS may be assessed with variability in the time post injury, as well as the circumstances under which the assessment is performed. Of note, several studies show that GCS is unreliable as a severity measure [16]. Additionally, the increasingly widespread use of early intubation and sedation during transport further complicates interpretation of the GCS [17]. GCS or other corresponding tools should be seen as methods to monitor the level of consciousness and to transmit this information during the process of care, and not to label the clinical condition as a whole. Although GCS is monitored in patients with brain hypoxia due to cardiac arrest, it is not used to assess the severity or prognosis of the condition, because there is a more exact measure available, which is directly related to the underlying pathophysiology (= ROSC i.e., return of spontaneous circulation).

### 3.2. Post-Traumatic Amnesia

Numerous studies have shown that PTA is the best clinical predictor of long-term cognitive outcome after a TBI [18]. PTA is characterized by variable impairments of cognition including memory and attention, confusion, excessive sleepiness, restlessness and agitation [19]. Surprisingly, the pathophysiological basis for PTA is still poorly understood, although recent work provides evidence that PTA is caused by a transient disconnection between parts of the limbic system involved in memory encoding, in particular a disruption in the functional connectivity between the medial temporal lobes and other parts of the default mode network that resolves with the emergence from PTA [20]. The severity and location of diffuse axonal injury (DAI) may be important in producing this disconnection, as white matter damage within the cingulum connections of the parahippocampal gyrus are associated with prolonged PTA duration [20]. Dysfunction within the frontal lobes is also likely to contribute to PTA, possibly reflecting a transient global disruption of functional connectivity [21]. How various acute disconnections contribute to the acute clinical symptoms and associate with the outcome, is poorly understood.

There are problems with the clinical assessment and interpretation of PTA. Very few studies have compared the reliability and reproducibility of different tools to measure PTA. Clinical estimates of PTA are often done retrospectively, but these may be inaccurate due to recall bias [22]. These retrospective assessments may give both longer and shorter estimates for PTA than prospective evaluations [23]. In addition, there are different severity classifications also based on the length of PTA, some classifying 1–24 h of PTA [24] and some >24 h [2] as indicative of moderate TBI.

Perhaps unsurprisingly, there is often poor concordance between GCS and PTA. Many patients with TBI who might by GCS criteria alone be considered mild have prolonged PTA durations, indicating a more severe injury [25,26]. These two tools provide complementary information about brain function and often lead to quite different estimates of clinical severity. Failure to assess either of these accurately and in a standardized fashion may be a major contributor to disparate and often inaccurate severity classification and prognosis after a TBI. These challenges have probably significantly influenced the very heterogeneous results in clinical outcome studies.

### 3.3. Confounders for the Use of GCS and PTA to Assess Injury Severity

The assessment of TBI severity in the acute care setting is often confounded by difficulties collecting or interpreting the GCS or PTA (Table 2). These include language issues, inexperienced evaluators, drug effects on level of consciousness and retrospective bias among other things. These confounders often lead to skewed estimates of TBI severity in either direction. For example, drowsiness, confusion, and amnesia might be attributed to intoxication leading to an underestimation of TBI severity, or vice versa. The potential importance of these confounders for long-term outcome may only become apparent after the acute period. How the confounders have possibly influenced the acute assessment is usually impossible to determine reliably afterwards.

A patient who arrives unconscious and diagnosed with severe TBI may regain consciousness rapidly and recover quickly, especially in the setting of clinical confounders such as alcohol intoxication. A person who fell on the ground, hitting their head and convulsing immediately, may be deeply unconscious for a while but recover rapidly, if the lowered consciousness was actually post-ictal and not caused by the brain trauma. Recognition of such confounders is not straightforward, and seldom are the initial severity assessments corrected to account for erroneous classification after the fact.

## 4. Tools for Predicting Long-Term Outcome after TBI

Variability in long-term outcomes is partly caused by distinct types of TBI, which are not captured by current global injury severity classification schemes. For example, epidural hematomas are life threatening acutely but rapid treatment often produces very good outcomes. In contrast, DAI may be underestimated in the acute setting giving an impression of a mild injury but is often associated with long-term disability. Although local visible traumatic lesions may produce recognizable symptoms, the overall outcome is mostly dependent on extent and severity of diffuse damage in the brain networks [27], which is largely invisible for routine clinical imaging [28]. This is especially important because the external forces causing TBI frequently cause several different injury types within the same brain. Ideally, the uncertainty in predicting long-term outcomes that results from pathophysiological uncertainty should be reflected in the injury classification, so that inappropriate and potentially nihilistic or overly optimistic clinical decision-making can be avoided.

Quantifying long-term outcome after TBI is a complex issue, as it is influenced by multiple factors that have variable causal relationships with the TBI itself. These include genetic, biologic, psychological, and social factors. If quality of life is used as an outcome, paradoxically those with severe TBI often experience better quality of life than those with milder injuries [29]. Although this paradox largely stems from reduced self-awareness, this highlights the uncertainties we currently face when using the very obscure “acute severity” as a predictor of TBI outcome, which is at least as ill-defined, subjective, and context dependent as is the acute severity. In other words, because both the acute severity of TBI and outcome from TBI are often inaccurate and multifaceted concepts, it is unsurprising that research has produced such different results on the association between these two.

Current approaches to predicting long-term outcome after TBI are also limited in their accuracy. There are a range of tools for predicting outcomes after TBI, which use a multivariate approach to combining many factors that potentially influence outcomes, e.g., CRASH-model and IMPACT-calculator [30,31]. Variables assessed include age, pupillary reactivity, presence of secondary injuries (esp. hypoxia/hypovolemia), comorbidities, and brain imaging findings. By combining different predictors, emerging assessment tools have achieved superior predictive value in patients with moderate or severe TBI [32]. The complexity of assessing TBI is highlighted by the fact that even by using combination models, we are able to explain only 35% of the variance outcome after severe TBI [33]. Multivariate models derived from large study populations perform fairly well (80–90% accuracy) in predicting mortality or poor outcome, but outcomes are much more complex than being dead or severely disabled. New clinical tools are needed for evaluation of injuries at both ends of the TBI spectrum. In case of mTBI (as well as TBI in general), factors such as pre-trauma cognitive achievement, personality traits, coping ability, resilience and availability of financial and social support systems have significant predictive value for outcome and quality of life [34,35]. Research has shown the current prediction algorithms for mTBI perform poorly [36] compared to prediction models for more severe cases (as discussed above).

## 5. Severity Is Not Stable

Acute evolution from an mTBI to fatal outcome is rare but well recognized and referred to as “walk/talk and die” clinical course. This is most commonly due to epidural or subdural hematomas, which can be managed surgically if diagnosed in a timely manner. Acute TBI, which appears mild at admission but takes a more severe course with a variable delay, can occur for multiple reasons, usually as consequence of some type of secondary injury. Moreover, patients who have had prior TBIs, such as those who have sustained repeated injuries while participating in contact sports, are less likely to recover fully from an index TBI [37].

The concept of a single mTBI as an always-innocent incident has undergone reassessment as research has shown their potential long-term sequelae [38], as well as the common finding of suboptimal recovery from mTBI [3]. Recent research suggests that repeated head impacts even in the absence of clinical signs of brain injury may lead to permanent alterations of brain structure and function, and in some cases, trigger long-term neurological sequelae [8]. However, most patients who sustain an mTBI return to premorbid levels of function within days to weeks post-injury. It should also be recognized that risk factors for poor outcome change over time. For example, during the acute stage after injury, recovery is primarily driven by factors related to brain physiology, while at later stages other factors are also determinative, including psychosocial, economic, environmental issues, personality, coping mechanisms, and medical comorbidities. Pediatric TBI is a special problem, because the injury disrupts a developing brain. Thus, the consequences may not be discernible before adulthood, as shown in follow-up studies [39]. The concept of ‘severity’ in pediatric TBI may be even more obscure than in adult TBI, and the same holds true for TBIs in elderly people.

Additionally, emerging evidence indicates that TBI when mild and recurrent or more severe can result in slowly progressive neurodegeneration [40], although the mechanisms underlying such neurological decline are still poorly understood. TBI resulting in life long neurological impairment is best considered as a chronic life condition and not as an isolated singular event.

## 6. Are We Fooling Ourselves?

As shown above, the concept of severity is poorly defined and may be used in variable contexts. Furthermore, descriptors of clinical outcome depend heavily on the measures and classifiers incorporated in the evaluation instruments [41]. Different pathophysiological processes should be graded in a consistent manner, based on accurately measurable, mechanistically relevant injury biomarkers using imaging, physiological, and molecular techniques. Brain injury medicine has much to learn from fields such as oncology, where different cancers have been accurately defined and differentiated based on molecular biology, quantifiable clinical variables, and objective pathological markers. The complexity and heterogeneity of TBI requires comparative approaches before we can expect true advances in treatment. The problems raised in this paper have recently been discussed also in relation to experimental TBI research, highlighting the fact that TBI severity may be very different in experimental animals and humans [42]. Given all the complexities, we are fooling ourselves and everyone else by having such a definitive classification system that does not acknowledge the tremendous uncertainty about long-term outcomes. In addition, by giving uncertain and thus potentially misleading labels of injury severity, clinicians may unwittingly complicate and trigger judicial process by creating a seeming discrepancy between initial severity and outcome, and in doing so, may increase stress and suffering for the patient, their families, and society at large. Table 3 lists potential consequences of failed severity estimates.

Current international efforts within the InTBIR (International Traumatic Brain Injury Research Initiative) collaboration [43], such as CENTER-TBI and TRACK-TBI, will undoubtedly improve assessment of acute TBIs and the contribution of different factors that affect clinical outcome, as already published [44]. Yet, the challenges outlined in this paper will likely face us for many years to come due to a variety of factors including competing interests across different sectors of society (medical, ethical, legal, political), nomenclature biases including entrenchment of use of certain severity labels, as well as the complexity of TBI and the lack of widely available reliable biomarker panels and MRI techniques.

## 7. A Proposal: From Severity Labels to Risk Assessment over Time?

Due to our capability to assess the true severity of TBI being often suboptimal, it is imperative that we find better and more appropriate ways to classify these patients. We suggest that instead of severity, we should consider using “risk-labels”. This approach is well accepted in oncology both by the professionals and patients, acknowledging that certain types within organ-specific malignancies have better response for targeted treatments and prognosis than others, but there still are risks for either good or bad outcomes. Likewise, instead of categorizing adjectives mild, moderate, or severe TBI, we propose using the terms “low-risk TBI”, “medium-risk TBI”, and “high-risk TBI” (in more detail below). Adoption of a classification system that acknowledges uncertainty would more accurately reflect clinical reality, since in many cases of mTBI there is a lower risk of a complicated clinical course or poor neurological outcome, but the terminology recognizes that such risk exists. Similarly, “high-risk TBI” acknowledges that while there is considerable risk for death or severe disability, sometimes these risks do not materialize, and many patients make very gratifying recoveries. Both for those who do not recover from mTBI and for those who fully recover from a severe TBI, adoption of these clinical classifiers would be a much more comprehensible means to convey clinical expectation to those injured, their families, and caretakers, including medical and allied health professionals. It is misleading to patients and families to refer to a mild TBI, which nonetheless resulted in permanent incapacity for work. It is equally misleading to use this term when referring to the injury to workmates, employers, and insurance carriers. On the other end of the spectrum, patients who suffer a severe TBI, but recover without any alterations in well-being or functioning, may sometimes experience unnecessary stigma and adverse consequences in employment and social relationships. In worst cases, patients with very low level of consciousness in the acute stage may be deemed unsalvageable without sufficient certainty and thus withheld from necessary treatments, although some of the most severe cases may show a remarkable recovery with time.

While we are fully aware that, based on current knowledge, the proposed risk-grading relies mainly in the same variables as the current severity classification, there is a fundamental difference in the message given both for the patients, proxies, and medical professionals. Instead of current labels—mild, complicated mild, moderate, severe, very severe—we could develop improved grading and staging of TBIs, based on the available large recent multidimensional databases from the InTBIR efforts. This kind of change would better depict the large variability of TBIs, give better comparable tools for clinical research, and avoid causing unnecessary harm from misleading labels. Future TBI severity classifications should not rely solely on clinical information, for the reasons discussed above. Severity classification should incorporate objective measures of pathophysiology such as neuroimaging and fluid biomarkers [45,46,47].

A novel risk-based grading has to be based on more reliable and measurable variables than the current severity classification. It has to give basis for targeted treatment interventions, allowing better comparison of treatment options for well-defined TBI sub-populations. TBI pathophysiological heterogeneity must be considered without grouping distinct traumatic lesions under the same categorical umbrella. The dynamic nature of TBI recovery and variability inherent in same must also be accounted for as these may change over time relative to risk grading. Such a grading system would also have to acknowledge that there are two major types of risks in TBI: a risk for acute serious course, and a risk for long-lasting or permanent problems, and that these are separate from each other. Epidural hematoma is a good example: if the treatment is severely delayed or not given at all, it may well rapidly lead to a fatal end, but if treated adequately in time the risk for long-term problems is low. Building a new, widely accepted classification for TBI is a significant effort, but different stakeholders should aim at developing it as an international consensus undertaking. In Table 4, we outline what kind of factors could be included in a new risk-based classification. Analyzing large existing well-characterized databases using artificial intelligence and machine learning should build the basis for such risk assessments. Due to the complexity of TBI and the multiple pre-injury, injury and post-injury factors that may influence outcome especially over the long-term, a new classification should be able to separate patient-related risk factors (upper row in Table 4) from injury- and treatment-related (other rows in Table 4). The rationale behind this is to keep the injury-related pathophysiological risk separate from risks that are related to person history or current life-situation, because the interventions required are very different. How this would be best done requires careful analytics of existing data and international agreements, since there are some factors that will be challenging to deal with in a rigorous classification system, such as the influence of genetics.

## 8. Conclusions

We recommend that the clinical and scientific TBI community stop using severity labels based on features apparent at hospital admission, which may cause more confusion than clarity for patients, families, and society at large. At the very least, the severe shortcomings of the current grading should be clearly and widely recognized. Consideration should be given to utilization of risk assessments over time that more adequately tap into the information that we as clinicians, patients, and their families need to provide data driven prognoses, care, and education. There is a need for an international consensus on how risk assessments should be defined, and on how imaging, physiologic, and molecular biomarkers can be incorporated into such assessments, as ongoing research refines their prognostic and predictive value. 

## Figures and Tables

**Table 1:** Different viewpoints on TBI severity.

Acute risk of death/mortality
Quantifying brain biomarker efflux
Risk/need for neurosurgical measures
Level/intensity of treatment needed (home, ED ^1^, ward, ICU ^2^)
Duration of hospital stay
Outcome at the end of hospital care
Quantification of brain tissue injury
Functional/vocational recovery
Independency as outcome
Subjective symptoms as outcome
Quality of life as outcome

^1^ Emergency department, ^2^ Intensive care unit.

**Table 2:** Confounders of assessing TBI severity (in alphabetical order).

Confounders for GCS ^1^	Confounders for PTA ^2^
CNS-active medications (sedatives, opiates) Hearing deficits Hypovolemia Hypoxia Inebriation (alcohol, drugs), intoxication Language problems Orbital injuries Psychic shock Seizures Sensory/motor loss (hemiparesis, SCI ^3^) Sleep deprivation Surgical measures	CNS-active medications (sedatives, opiates) False memories Inebriation (alcohol, drugs), intoxication Islands of memory ^4^ Language problems Psychic shock Psychogenic amnesia Sleep Surgical measures

^1^ Glasgow Coma Score, ^2^ post-traumatic amnesia, ^3^ spinal cord injury. ^4^ Islands of memory are remembrances within PTA and often the duration of PTA is estimated to the first islands of memory, although the recovery of continuous memory (= definition of recovery from PTA) may have taken place much later.

**Table 3:** Potential negative consequences of wrong estimates of TBI severity.

	Underestimation	Overestimation
Patient	Potentially fatal injuries remain undetected (e.g., EDH ^1^) Secondary injuries remain untreated Risk for new injuries due to TBI-related problems Returning to work/play too early, prolonged recovery Secondary psychiatric problems due to poor performance (burnout, depression) Social problems due to misunderstood poor performance or neuropsychiatric symptoms Firing from work, economical losses Lack of insurance compensation, litigation	Treatments withheld due to pessimistic prognosis Risk for overtreatment and hazardous measures Unnecessary hospital care and examinations Economical losses due to prolonged sick leave Non-TBI related disorders remain undiagnosed and untreated Psychosocial sequelae due to wrong label, unnecessary psychic stress for the proxies Loss of self-esteem, fear of future Adoption of unnecessary illness behavior
Healthcare	Unnecessary new or secondary injuries Complex problems due to delayed diagnoses, loss of resources (in diagnostics and treatment) Involvement in litigation Lower confidence in healthcare	Waste of resources Poor treatment results due to wrong or overtreatment

^1^ epidural hematoma.

**Table 4:** An outline for new risk based TBI classification. The risks depend on the time from injury and available data.

	Acute Risk ^1^	Long-Term Risk ^2^
Patient-related	Age ^3^	Age ^3^
	Pre-injury somatic health	Pre-injury psychiatric or brain health
	Anticoagulant use	Earlier TBIs
	Genetics ^4^	Education
		Alcohol or drug abuse
		Psychological factors Physical activity
		Genetics ^4^
Injury-related	Other injuries ^5^	Other injuries ^5^
	Treatment delays	Injury details ^6^
Clinical assessments	Pupils	Duration of lowered consciousness
	GCS * (lowering-rising)	Duration of PTA ***
	Hypotension	Symptom severity ^7^
	Hypoxia	
	Elevated ICP **	
Treatment efforts	Craniotomy	Duration of hospital care
	Decompressive craniectomy	Patient education
	ICP lowering treatments ^8^	
Imaging	Type of lesions	Number of lesions
	Volume of intracranial bleeding	Depth of lesions
	Volume of contusions	Volume change ^9^
	Midline shift	DTI metrics ^10^
	Status of basal cisterns	
	Signs of herniation	
	Brainstem lesions	
Biomarkers^11^	Levels of glial markers	Levels of axonal markers
		Levels of neuronal markers
Complications	Seizures	Brain ischemia/hypoxia
	Brain ischemia	
	Serious infections	
	Cardiopulmonary complications	

^1^ Acute risk means risk of death or persistent vegetative stage (= GOSE^#^ 1–2). ^2^ Long-term risk means risk for permanent problems affecting daily life (= GOSE 3–7). ^3^ Age modifies several risks especially at both ends of the age spectrum. ^4^ Genetics probably affects acute and long-term risks differently, but available data is still very preliminary. ^5^ Assessed using standard tools such as Injury Severity Score or Abbreviated Injury Score. ^6^ Injuries sustained e.g., by violence or involving proxies have higher risk for chronic stress. ^7^ Symptom severity both acutely and at e.g., two weeks shown to be predictive for outcome. ^8^ Use of 1., 2., and 3. tier therapies describe the severity of the ICP-problem. ^9^ Requires serial imaging and validated volumetric tools. ^10^ Optimal time-point and methodology still uncertain, requires standardization. ^11^ Protein, metabolomics, and microRNA biomarkers and combinations of biomarkers as future options. * Glasgow Coma Score, ** intracranial pressure, *** post-traumatic amnesia, ^#^ Glasgow Outcome Scale Extended.

## Data Availability

Not applicable.

## References

[1] Saatman K.E., Duhaime A.C., Bullock R., Maas A.I., Valadka A., Manley G.T. (2008). Classification of traumatic brain injury for targeted therapies. J. Neurotrauma.

[2] Head Injury Interdisciplinary Special Interest Group of the American Congress of Rehabilitation Medicine (1993). Definition of mild traumatic brain injury. J. Head Trauma Rehabil..

[3] Nelson L.D., Temkin N.R., Dikmen S., Barber J., Giacino J.T., Yuh E., Levin H.S., McCrea M.A., Stein M.B., Mukherjee P. (2019). Recovery After Mild Traumatic Brain Injury in Patients Presenting to US Level I Trauma Centers: A Transforming Research and Clinical Knowledge in Traumatic Brain Injury (TRACK-TBI) Study. JAMA Neurol..

[4] Coronado V.G., McGuire L.C., Faul M., Sugerman D.E., Pearson W.S., Zasler N.D., Katz D.I., Zafonte R.D. (2013). Traumatic brain injury epidemiology and public health issues. Brain Injury Medicine.

[5] Te Ao B., Brown P., Tobias M., Ameratunga S., Barker-Collo S., Theadom A., McPherson K., Starkey N., Dowell A., Jones K. (2014). Cost of traumatic brain injury in New Zealand: Evidence from a population-based study. Neurology.

[6] Carroll L.J., Cassidy J.D., Holm L., Kraus J., Coronado V.G. (2004). Methodological issues and research recommendations for mild traumatic brain injury: The WHO Collaborating Centre Task Force on Mild Traumatic Brain Injury. J. Rehabil. Med..

[7] Sharp D.J., Jenkins P.O. (2015). Concussion is confusing us all. Pract. Neurol..

[8] Tagge C.A., Fisher A.M., Minaeva O.V., Gaudreau-Balderrama A., Moncaster J.A., Zhang X.-L., Wojnarowicz M.W., Casey N., Lu H., Kokiko-Cochran O.N. (2018). Concussion, microvascular injury, and early tauopathy in young athletes after impact head injury and an impact concussion mouse model. Brain.

[9] McCrory P., Feddermann-Demont N., Dvořák J., Cassidy J.D., McIntosh A., Vos P.E., Echemendia R.J., Meeuwisse W., Tarnutzer A.A. (2017). What is the definition of sports-related concussion: A systematic review. Br. J. Sports Med..

[10] Levin H.S., O’Donnell V.M., Grossman R.G. (1979). The Galveston Orientation and Amnesia Test: A practical scale to assess cognition after head injury. J. Nerv. Ment. Dis..

[11] Meares S., Shores E.A., Taylor A.J., Lammél A., Batchelor J. (2011). Validation of the Abbreviated Westmead Post-traumatic Amnesia Scale: A brief measure to identify acute cognitive impairment in mild traumatic brain injury. Brain Inj..

[12] Zuercher M., Ummenhofer W., Baltussen A., Walder B. (2009). The use of Glasgow Coma Scale in injury assessment: A critical review. Brain Inj..

[13] King N.S., Crawford S., Wenden F.J., Moss N.E., Wade D.T., Caldwell F.E. (1997). Measurement of post-traumatic amnesia: How reliable is it?. J. Neurol. Neurosurg. Psychiatry.

[14] Giacino J.T., Fins J.J., Laureys S., Schiff N.D. (2014). Disorders of consciousness after acquired brain injury: The state of the science. Nat. Rev. Neurol..

[15] Foreman B.P., Caesar R.R., Parks J., Madden C., Gentilello L.M., Shafi S., Carlile M.C., Harper C.R., Diaz-Arrastia R.R. (2007). Usefulness of the abbreviated injury score and the injury severity score in comparison to the Glasgow Coma Scale in predicting outcome after traumatic brain injury. J. Trauma.

[16] Perrin P.B., Niemeier J.P., Mougeot J.L., Vannoy C.H., Hirsch M.A., Watts J.A., Rossman W., Grafton L.M., Guerrier T.D., Pershad R. (2015). Measures of injury severity and prediction of acute traumatic brain injury outcomes. J. Head Trauma Rehabil..

[17] Barker M.D., Whyte J., Pretz C.R., Sherer M., Temkin N., Hammond F.M., Saad Z., Novack T. (2014). Application and clinical utility of the Glasgow Coma Scale over time: A study employing the NIDRR traumatic brain injury model systems database. J. Head Trauma Rehabil..

[18] Königs M., de Kieviet J.F., Oosterlaan J. (2012). Post-traumatic amnesia predicts intelligence impairment following traumatic brain injury: A meta-analysis. J. Neurol. Neurosurg. Psychiatry.

[19] Marshman L.A., Jakabek D., Hennessy M., Quirk F., Guazzo E.P. (2013). Post-traumatic amnesia. J. Clin. Neurosci..

[20] De Simoni S., Grover P.J., Jenkins P.O., Honeyfield L., Quest R.A., Ross E., Scott G., Wilson M.H., Majewska P., Waldman A.D. (2016). Disconnection between the default mode network and medial temporal lobes in post-traumatic amnesia. Brain.

[21] Metting Z., Rödiger L.A., de Jong B.M., Stewart R.E., Kremer B.P., van der Naalt J. (2010). Acute cerebral perfusion CT abnormalities associated with posttraumatic amnesia in mild head injury. J. Neurotrauma.

[22] Friedland D., Swash M. (2016). Post-traumatic amnesia and confusional state: Hazards of retrospective assessment. J. Neurol. Neurosurg. Psychiatry.

[23] Roberts C.M., Spitz G., Ponsford J.L. (2016). Comparing Prospectively Recorded Posttraumatic Amnesia Duration with Retrospective Accounts. J. Head Trauma Rehabil..

[24] Greenwood R. (2002). Head injury for neurologists. J. Neurol. Neurosurg. Psychiatry.

[25] Dikmen S., Machamer J., Temkin N. (2001). Mild head injury: Facts and artifacts. J. Clin. Exp. Neuropsychol..

[26] Cota M., Moses A., Jikaria N., Bittner K.C., Diaz-Arrastia R.R., Latour L.L., Turtzo L.C. (2019). Discordance between Documented Criteria and Documented Diagnosis of Traumatic Brain Injury in the Emergency Department. J. Neurotrauma.

[27] Van Eijck M.M., Schoonman G.G., van der Naalt J., de Vries J., Roks G. (2018). Diffuse axonal injury after traumatic brain injury is a prognostic factor for functional outcome: A systematic review and meta-analysis. Brain Inj..

[28] Amyot F., Arciniegas D.B., Brazaitis M.P., Curley K.C., Diaz-Arrastia R., Gandjbakhche A., Herscovitch P., Hinds S.R., Manley G.T., Pacifico A. (2015). A Review of the Effectiveness of Neuroimaging Modalities for the Detection of Traumatic Brain Injury. J. Neurotrauma.

[29] Sasse N., Gibbons H., Wilson L., Martinez-Olivera R., Schmidt H., Hasselhorn M., von Wild K., von Steinbüchel N. (2013). Self-awareness and health-related quality of life after traumatic brain injury. J. Head Trauma Rehabil..

[30] Perel P., Arango M., Clayton T., Edwards P., Komolafe E., Poccock S., Roberts I., Shakur H., Steyerberg E., MRC CRASH Trial Collaborators (2008). Predicting outcome after traumatic brain injury: Practical prognostic models based on large cohort of international patients. BMJ.

[31] Steyerberg E.W., Mushkudiani N., Perel P., Butcher I., Lu J., McHugh G.S., Murray G.D., Marmarou A., Roberts I., Habbeba J.D. (2008). Predicting outcome after traumatic brain injury: Development and international validation of prognostic scores based on admission characteristics. PLoS Med..

[32] Roozenbeek B., Lingsma H.F., Lecky F.E., Lu J., Weir J., Butcher I., McHugh G.S., Murray G.D., Perel P., Maas A.I. (2012). Prediction of outcome after moderate and severe traumatic brain injury: External validation of the International Mission on Prognosis and Analysis of Clinical Trials (IMPACT) and Corticoid Randomisation After Significant Head injury (CRASH) prognostic models. Crit. Care Med..

[33] Maas A.I., Lingsma H.F., Roozenbeek B. (2015). Predicting outcome after traumatic brain injury. Handb. Clin. Neurol..

[34] Van der Naalt J., Timmerman M.E., de Koning M.E., van der Horn H.J., Scheenen M.E., Jacobs B., Hageman G., Yilmaz T., Roks G., Spikman J.M. (2017). Early predictors of outcome after mild traumatic brain injury (UPFRONT): An observational cohort study. Lancet Neurol..

[35] Iverson G., Silverberg N., Lange R.T., Zasler N.D., Zasler N.D., Katz D.I., Zafonte R.D. (2013). Conceptualizing Outcome From Mild Traumatic Brain Injury. Brain Injury Medicine.

[36] Cnossen M.C., van der Naalt J., Spikman J.M., Nieboer D., Yue J.K., Winkler E.A., Manley G.T., von Steinbüchel N., Polinder S., Steyerberg E.W. (2018). Prediction of Persistent Post-Concussion Symptoms Following Mild Traumatic Brain Injury. J. Neurotrauma.

[37] Dams-O’Connor K., Spielman L., Singh A., Gordon W.A., Lingsma H.F., Maas A.I., Manley G.T., Mukherjee P., Okonkwo D.O., Puccio A.M. (2013). The impact of previous traumatic brain injury on health and functioning: A TRACK-TBI study. J. Neurotrauma.

[38] Henry L.C., Tremblay S., De Beaumont L. (2017). Long-Term Effects of Sports Concussions: Bridging the Neurocognitive Repercussions of the Injury with the Newest Neuroimaging Data. Neuroscientist.

[39] Sariaslan A., Sharp D.J., D’Onofrio B.M., Larsson H., Fazel S. (2016). Long-Term Outcomes Associated with Traumatic Brain Injury in Childhood and Adolescence: A Nationwide Swedish Cohort Study of a Wide Range of Medical and Social Outcomes. PLoS Med..

[40] Wilson L., Stewart W., Dams-O’Connor K., Diaz-Arrastia R., Horton L., Menon D.K., Polinder S. (2017). The chronic and evolving neurological consequences of traumatic brain injury. Lancet Neurol..

[41] Voormolen D.C., Cnossen M.C., Polinder S., von Steinbuechel N., Vos P.E., Haagsma J.A. (2018). Divergent Classification Methods of Post-Concussion Syndrome after Mild Traumatic Brain Injury: Prevalence Rates, Risk Factors, and Functional Outcome. J. Neurotrauma.

[42] Yamamoto S., Levin H.S., Prough D.S. (2018). Mild, moderate and severe: Terminology implications for clinical and experimental traumatic brain injury. Curr. Opin. Neurol..

[43] Tosetti P., Hicks R.R., Theriault E., Phillips A., Koroshetz W., Draghia-Akli R. (2013). Toward an international initiative for traumatic brain injury research. J. Neurotrauma.

[44] Gravesteijn B., Sewalt C., Ercole A., Akerlund C., Nelson D., Maas A.I.R., Menon D., Lingsma H.F., Steyerberg E.W. (2020). Toward a new multidimensional classification of traumatic brain injury: A CENTER-TBI study. J. Neurotrauma.

[45] Majdan M., Brazinova A., Rusnak M., Leitgeb J. (2017). Outcome Prediction after Traumatic Brain Injury: Comparison of the Performance of Routinely Used Severity Scores and Multivariable Prognostic Models. J. Neurosci. Rural Pract..

[46] Orešič M., Posti J.P., Kamstrup-Nielsen M.H., Takala R.S.K., Lingsma H.F., Mattila I., Jäntti S., Katila A.J., Carpenter K.L.H., Ala-Seppälä H. (2016). Human Serum Metabolites Associate with Severity and Patient Outcomes in Traumatic Brain Injury. EBioMedicine.

[47] Thelin E., Al Nimer F., Frostell A., Zetterberg H., Blennow K., Nyström H., Svensson M., Bellander B.M., Piehl F., Nelson D.W. (2019). A serum protein biomarker panel improves outcome prediction in human traumatic brain injury. J. Neurotrauma.

